# Single arm NCRI phase II study of CHOP in combination with Ofatumumab in induction and maintenance for patients with newly diagnosed Richter’s syndrome

**DOI:** 10.1186/s12885-015-1048-9

**Published:** 2015-02-13

**Authors:** Toby A Eyre, Ruth Clifford, Corran Roberts, Lucy Boyle, Anne Francis, Anna Schuh, Susan J Dutton

**Affiliations:** 1 Department of Haematology & The Early Phase Clinical Trial Unit, Oxford University Hospitals NHS Trust, Churchill Hospital, Oxford, OX3 7EJ UK; 2 NIHR BRC Oxford Molecular Diagnostic Centre, Oxford University Hospitals NHS Trust, John Radcliffe Hospital, Oxford, OX3 9DU UK; 3 Centre for Statistics in Medicine (CSM), Nuffield Department of Orthopaedics, Rheumatology and Musculoskeletal Sciences, University of Oxford, The Botnar Research Centre, Windmill Road, Oxford, OX3 7LD UK; 4 CHOP-OR Trial Office, OCTO - Oncology Clinical Trials Office, Department of Oncology, University of Oxford, Old Road Campus Research Building, Roosevelt Drive, Oxford, OX3 7DQ UK; 5 Oxford Clinical Trials Research Unit & Centre for Statistics in Medicine, Nuffield Department of Orthopaedics, Rheumatology and Musculoskeletal Sciences, The Botnar Research Centre, University of Oxford, Windmill Road Oxford, OX3 7LD UK

**Keywords:** Ofatumumab, CHOP, TP53, Richter’s syndrome, Chronic lymphocytic leukaemia, Diffuse large B cell lymphoma, Phase II, Rare cancers

## Abstract

**Background:**

Transformation of B-cell chronic lymphocytic leukaemia (B-CLL) to diffuse large B cell lymphoma (DLBCL) (Richter’s syndrome (RS)) is a rare (2-15% of patients) but catastrophic complication of B-CLL. Dose-intense chemotherapy regimens investigated in small single institution trials, but with the exception of bone marrow transplantation for a minority of patients, little has improved the median overall survival of patients with RS beyond eight months. Patients are often elderly, immunosuppressed, possess co-morbidities and have a deteriorating performance status. TP53 disruption is a common molecular abnormality noted in RS and contributes to the tumour’s chemotherapy resistance. Ofatumumab is a fully human anti-CD20 monoclonal IgG1κ antibody that targets a unique epitope on B lymphocytes. It has displayed increased binding affinity and a longer dissociation time when compared to rituximab resulting in improved complement dependent cellular cytotoxicity (CDCC); a mechanism with the potential to overcome apoptosis-resistance in TP53 disruption. Given the prevalence of TP53 disruption in RS, Ofatumumab was considered a relatively non-toxic agent with a sound rationale to test in a prospective multicentre trial as an adjunct to CHOP induction and subsequent ofatumumab maintenance therapy in responding patients.

**Methods/Design:**

The CHOP-OR study is a prospective phase II study to evaluate the safety, feasibility and activity of a CHOP chemotherapy in combination with ofatumumab in induction and subsequent maintenance for patients with newly diagnosed RS. The primary objective will be the overall response rate (ORR) in patients with RS after six cycles of CHOP-O. The secondary objectives include feasibility of recruitment, progression free survival (PFS), overall survival (OS) and toxicity. The study will be accompanied by exploratory analysis of the genomic landscape of RS in newly diagnosed patients.

**Discussion:**

The CHOP-OR trial evaluates the safety, feasibility and activity of CHOP plus Ofatumumab induction and Ofatumumab maintenance in new RS patients. The study is currently recruiting and has met the interim analysis criteria, with more than 7 of the first 25 participants achieving a CR or PR after six cycles of CHOP-O. The study has the potential to identify predictive biomarkers for this treatment modality.

**Trial registration:**

NCT01171378.

## Background

B-CLL is the most prevalent chronic lymphoproliferative disease and follows a typically indolent course, however 2-15% of patients [1-3] transform to an invariably aggressive, chemo-resistant high-grade non-Hodgkin’s lymphoma (NHL) [4]. This high-grade transformation of CLL was first recognised in 1928 by Maurice Richter who described an aggressive, life-threatening presentation of rapidly fatal generalized lymphadenopathy and hepatosplenomegaly [4] that he called a “reticular cell sarcoma” arising in a patient with B-CLL. Whereas all subtypes of NHL taken together represent the fifth most common cancer type in the world and its incidence continues to increase [5], Richter’s syndrome (RS) is rare and characterised by disproportionate weight loss, rapidly growing and/or asymmetrical lymphadenopathy or extranodal fluorodeoxyglucose ([^18^F] FDG) positron-emission-tomography (PET) CT-avid masses, new B symptoms, a rapidly rising lactate dehydrogenase (LDH), or new hypercalcaemia in a patient with known B-CLL. The vast majority of RS represent transformations to an activated B-cell type (ABC) diffuse large B-cell lymphoma (DLBCL) (90-95%) with a small proportion transforming to Hodgkin’s lymphoma [6]. The prognosis of *de novo* DLBCL has much improved over the last 15 years with the introduction of the anti-CD20 monoclonal antibody rituximab to an anthracycline based regimen, typically CHOP (cyclophosphamide, doxorubicin, oncovin (vincristine), prednisolone) such that the long term survival in those fit for anthracycline-based therapy is approaching 70% [7]. Unfortunately, the same cannot be said for DLBCL-Richter’s syndrome. RS can present in either heavily pre-treated, immunosuppressed or in untreated B-CLL patients [8]. Patients typically present with a deteriorating performance status. The median age of B-CLL diagnosis is 72 years [9], and therefore patients often possess dose-limiting co-morbidities. Given its rarity, no multicentre randomised controlled trials have been performed. Alkylator, anthracycline, platinum and purine analogue chemotherapy have formed the backbone of a number of regimens trialled in RS. R-CHOP (cyclophosphamide, doxorubicin, vincristine, and prednisone with rituximab) [7], R-hyper-CVXD-MA [10] (fractionated cyclophosphamide, vincristine, liposomal daunorubicin, and dexamethasone plus rituximab and alternating with methotrexate and cytarabine with rituximab), hyper-CVXD alone [11], FACPGM (fludarabine, cytarabine, cyclophosphamide, cisplatin and GM-CSF) [12], OFAR1 [13] and OFAR2 [14] (oxaliplatin, fludarabine, cytarabine, rituximab and pegfilgrastim) [2] treatment regimens have been used. The best response rates are 41% with hyper-CVXD and R-hyper-CVXD-MA and 50% with OFAR1, although responses are short-lived. Moreover, these regimens are toxic and inappropriate for many patients with RS. The median survival of 8–10 months [2] from diagnosis has generally not been bettered in the literature. Recent published data using R-CHOP in 15 patients with Richter’s Syndrome showed an overall response rate of 67% but the progression free survival (10 months) and overall survival (21 months), although an improvement, was still short and the study number was very small [15]. Autologous and allogeneic bone marrow transplantation are reserved for younger patients with good performance status [8].

Ofatumumab is a fully humanised monoclonal IgG anti-CD20 antibody. It specifically and powerfully targets a unique CD20 epitope on B cells. When compared with rituximab, it binds with increased affinity and has a longer dissociation time, both of which improves its complement-mediated cellular cytotoxicity [16,17]. As a result, ofatumumab has a greater potential to induce B cell apoptosis independently of p53 than rituximab, and has been shown to be efficacious and non-toxic in relapsed B-CLL refractory to fludarabine and alemtuzumab; a group that commonly possess TP53 mutations and/or deletions [18]. Given the high incidence of TP53 disruption in patients with DLBCL-RS [19] and that patients characteristically relapse early after initial response to induction, it was felt that ofatumumab as induction (alongside CHOP) and maintenance therapy would represent both a pragmatic and biologically-sound treatment for patients with RS within this phase II clinical trial. As many clinicians treat DLBCL-RS with R-CHOP outside of a clinical trial, the National Cancer Research Institute (NCRI) UK-based “CHOP-OR” single arm, multicentre study was therefore designed to investigate whether DLBCL-RS treated with six cycles of CHOP-O followed by maintenance ofatumumab every eight weeks for 48 weeks in responding patients was feasible and improved clinical efficacy over and above R-CHOP.

## Methods/Design

### Study design

The Study of CHOP with Ofatumumab in Patients with Richter’s Syndrome (CHOP-OR) trial is a single arm, multi-centre, non-randomised phase II NCRI feasibility study in 35 patients with newly diagnosed Richter’s Syndrome, recruiting from 10 centres in the UK since April 2011. Following eligibility confirmation, consent and then screening procedures, eligible patients will receive CHOP-O for six cycles, followed by six cycles of ofatumumab maintenance treatment every eight weeks, and then a three month follow-up period. The total duration of treatment and follow up for an individual participant is therefore 72 weeks; 20 weeks for induction/consolidation therapy, 40 weeks of maintenance, and 12 weeks of follow-up. The total duration of the trial is estimated to be four years. Any patients who withdraw from the study and are not evaluable will ideally be replaced, if within the recruitment period. It is possible that some clinicians will choose to perform autologous stem cell transplantation on responding patients prior to or following maintenance treatment. Data on outcome will be collected in these patients.

The primary objective of the CHOP-OR study is to determine objective response to Ofatumumab plus CHOP at week 20 as measured from start of treatment according to the Revised Response Criteria for Malignant Lymphoma (Cheson Criteria) [20]. Secondary objectives include assessing the feasibility of recruitment, progression free survival (PFS) and overall survival (OS), the clinical benefit and changes in patient-reported outcomes (PROs), safety and tolerability.

The CHOP-OR trial is an investigator initiated trial, and is sponsored by The University of Oxford. The trial has been designed through collaboration with the Oxford Clinical Trials Research Unit (OCTRU), the Oncology Clinical Trial Office (OCTO), the Centre for Statistics in Medicine (CSM) and the Department of Haematology and Oncology at the Oxford University Hospitals NHS Trust, in cooperation with the National Institute of Health and Research (NIHR) and the other participating trial sites. The trial is coordinated by OCTO who are responsible for the overall trial management, including; trial registration, all trial-related meetings, database management, monitoring reporting and quality assurance. Data collection is through electronic submissions of data by site staff via the web-based data capture system OpenClinica. Central and on-site monitoring is performed in accordance with Good Clinical Practice (GCP) guidelines throughout the trial. The centre for Statistics in Medicine (CSM), University of Oxford, will be responsible for all statistical analysis.

The final protocol was approved by the National Research Ethics Service (NRES) South Central – Oxford Committee, the Medicine and Healthcare products Regulatory Agency (MHRA) and by Research and Development departments of the other participating centres. The CHOP-OR trial is run in accordance with the principles of the Declaration of Helsinki (1996), UK Clinical Trials Regulations, the International Conference on Harmonisation guidelines of Good Clinical Practice (GCP) and the applicable policies of the sponsoring NHS Trust.

### Patient selection

To be eligible for inclusion in the CHOP-OR study, patients must be over 18 years of age with an ECOG Performance Status of 0–3, have a known diagnosis of B-CLL and a newly diagnosed, untreated and biopsy proven DLBCL Richter’s transformation according to the World Health Organization (WHO) classification. The histology results are to be reviewed locally in the first instance and for study inclusion. This is in an attempt to replicate the ‘real life’ scenario. The Biomedical Research Centre (BRC) Oxford Molecular Diagnostic Centre (MDC) will provide a central review of histopathology diagnostic samples during the study. Patients require a standard Computerised Tomography (CT) scan to be performed within 8 weeks prior to starting therapy. Patients will not be eligible if they have been previously treated with anthracycline-containing treatment for DLBCL within six months prior to registration, or have central nervous system involvement with RS or B-CLL, major medical co-morbidities, active and uncontrolled infection including HIV, active hepatitis B or C or other malignancy requiring active treatment (except treated basal cell carcinoma and non-invasive squamous cell carcinoma of the skin). Patients must use adequate contraception before entry and throughout the study if appropriate and women of childbearing potential must be willing to use adequate contraception during study and for 12 months after the last dose of ofatumumab. Adequate contraception is defined as abstinence, hormonal birth control or intrauterine devices. Pregnant patients or those breastfeeding are excluded, as well as those patients who are considered non-compliant with the CHOP-OR protocol, or those currently participating in any other interventional clinical study.

A total of 10 NHS Hospital Trusts in the UK are participating in this clinical trial. The centres are (listed alphabetically according to town or city): Queen Elizabeth Hospital, Birmingham; Royal Bournemouth Hospital, Bournemouth; Addenbrooke’s Hospital, Cambridge; St James’s University Hospital, Leeds; Royal Liverpool University Hospital, Liverpool; St Bartholomew’s Hospital, London; King’s College Hospital, London; The Christie Hospital, Manchester; Nottingham City Hospital, Nottingham; Churchill Hospital; Oxford.

### Informed consent

Written and verbal versions of the participant information sheet and informed consent will be presented to the participants by the attending investigator detailing no less than: the exact nature of the study; the implications and constraints of the protocol; the known side effects and any risks involved in taking part. It will be clearly stated that the participant is free to withdraw from the study at any time for any reason without prejudice to future care, and with no obligation to give the reason for withdrawal. The investigator will not undertake any study specific procedures until valid consent is obtained.

### Interventions

All patients will be treated with up to a maximum of six three-weekly cycles of CHOP-O. This consists of: cyclophosphamide 750 mg/m^2^ intravenous (iv) bolus, doxorubicin 50 mg/m^2^ iv bolus, vincristine 1.4 mg/m^2^ (maximum 2 mg or 1 mg in patients over 70 years old) iv infusion in 50 ml sodium chloride 0.9% over 10 minutes, prednisolone 40 mg/m^2^ orally od, on day one and prednisolone 40 mg/ m2 orally od on days 2–5 of each cycle. Mesna prophylaxis is recommended in those with pre-existing bladder disorders. Ofatumumab 300 mg iv is given on day one of cycle one and 1000 mg iv is given on day 8 and 15 of cycle one as loading doses and then 1000 mg iv on day one of cycles 2–6. 1000 mg dosing was chosen on the basis of available pre-clinical [20] and clinical data [21] at the time of study design.

Maintenance ofatumumab 1000 mg iv is given every eight weeks for a maximum of 6 cycles, and started four weeks after day one, cycle six in those patients achieving at least a partial response according to the Cheson criteria after induction [22]. Ofatumumab infusion-related adverse effects such as allergic and anaphylactoid reactions are treated promptly as per the investigator’s discretion.

### Pre-treatment assessment

Potential participants with a new diagnosis of Richter’s transformation will receive a complete work-up which includes a CT scan of the neck, chest, abdominal and pelvis (NCAP), a bone marrow aspirate and trephine biopsy (BMAT) and routine haematology, biochemical and virology testing. In centres where it is feasible a PET CT is recommended at baseline. A CT NCAP is repeated after cycle six of CHOP-O and a BMAT repeated at this stage, at the end of week 72 and at progression if the bone marrow was involved at diagnosis with DLBCL. If patients decline treatment within the CHOP-OR trial, an adequate alternative treatment regimen, typically six cycles of CHOP-R without maintenance rituximab is offered. There is no availability of ofatumumab post-trial. Patients will be seen by their clinician according to standard care.

### Primary and secondary outcomes

The primary objective of the trial is to determine the objective response rate (ORR) of ofatumumab plus CHOP according to the Revised Response Criteria for Malignant Lymphoma (Cheson criteria) [22] as measured after induction. Response assessment is made after cycle six or if the patient withdraws from treatment earlier than cycle six, their response recorded at withdrawal will be used to determine the primary response. Or if an assessment is not performed at withdrawal, the latest disease response (e.g. post cycle four assessment) will be used. Patients will be classified as responders/non-responders as follows: complete remission (CR), partial remission (PR) or nodular partial remission (nPR) as responders while stable disease (SD) and progressive disease (PD) as non-responders. Evaluable patients are those who have received at least 1 full cycle of CHOP-O and had a response assessment (CT/PET scan or physical examination as appropriate). The secondary objectives are to demonstrate feasibility of recruitment in this rare disease, evaluate overall survival (OS), progression free survival (PFS), duration of response, time to next Richter’s therapy, constitutional symptoms (B-Symptoms), minimal residual disease (MRD), Eastern Cooperative Oncology Group (ECOG) performance status, patient reported outcome (PROs) and quality of life measures, safety, and toxicity. OS is defined in whole days as time from entry until death from any cause. Patients alive during the course of the trial will be censored at their last known follow-up date. PFS is defined in whole days as time from entry until lymphoma progression or death from any cause. Patients that are alive having not progressed during the course of the trial will be censored at their last known progression-free follow-up date. Duration of response is defined in whole days as the time between recorded response to disease progression or death from any cause. Patients who have not progressed or died will be censored at the date of their last follow-up visit at which the response was assessed. Time to next RS therapy is defined as the time from the end of study treatment and the start of the next RS therapy other than CHOP-O. Patients will be censored at the date of their last follow-up visit at which further treatment was assessed.

Constitutional symptoms (B symptoms) are defined as experiencing night sweats (in the absence of infection), unexplained unintentional weight loss ≥ 10% within the previous six months, extreme fatigue and recurrent and unexplained fever of >38°C for twice weeks, and will be evaluated at baseline and regular follow-ups. ECOG performance status will be summarised at baseline and regular follow-ups throughout the trial. Patient reported outcomes (PROs) will be evaluated using the European Organisation for Research and Treatment of Cancer (EORTC) QLQ-C30 and the EORTC QLQ-CLL16 at baseline and regular follow-up visits throughout the trial.

### Statistical design

#### Sample size determination and power

Simon’s minimax two-stage design was implemented to calculate the required sample size to identify a desired response of the trial drug of 47% with a power of 80% and significance of 20%. The study plans to accrue 25 patients in the first stage and 35 patients in total. The number of cycles of CHOP-O will depend on the assessment of activity and toxicity of the regimen by the investigator in each patient. If at least 16 responses are observed amongst 35 evaluable patients after six cycles of CHOP plus ofatumumab, efficacy will be considered to be equivalent or better than CHOP-R [7,10] and a phase II trial extension or a randomised study might be considered. The treatment will be considered ineffective if 33% or fewer patients achieve a response (PR or CR).

### Statistical analysis

The primary analysis will be performed on all patients recruited who received at least one full cycle of CHOP-O and had a response assessment by either clinical examination or CT scan. A sensitivity analysis will be performed using the per-protocol population defined as those patients not deviating from the protocol in such a manner that the assessment of efficacy endpoints may be biased. The safety population (all patients who receive at least one dose of the trial treatment) will be used to evaluate all safety endpoints. The primary analysis will report the proportion of responders after cycle six, including exact two-sided 95% confidence intervals. The analysis will also be reported at two further time points; after cycle four and at 72 weeks. OS, PFS, time to next Richter’s therapy and duration of response will be presented using Kaplan Meier plots and the time to event will be summarised using medians together with their two-sided 95% confidence intervals. Constitutional symptoms will be summarised at baseline and regular follow-ups throughout the trial. Patient reported outcomes will be presented using descriptive statistics for the EORTC pre-defined functions and scales at baseline and different follow-up time points. These will also be presented using longitudinal methods for individual patient outcomes. Frequency of number of adverse events (AEs) and the number of patients reporting AEs will be summarised overall and by system body class for SAEs, AEs with CTCAE grade ≥1, AEs with CTCAE grade ≥3 and AEs related to ofatumumab.

### Exploratory analysis

In addition to the safety and efficacy analysis, we will perform an exploratory analysis in this well annotated, prospectively collected patient data set. We will centrally review pathology on all patients and PET CT scan results within the preceding eight weeks of the start of study treatment. We are interested in correlating SUV max values with histopathological findings. Gene mutational analysis on all patients will be carried out on all patients and will correlate abnormalities found with clinical phenotype. Neither clonality data collected nor patient individual characteristics will be used to influence the treatment option for the patients prospectively.

### Monitoring committee

There is no Data and Safety Monitoring Committee for CHOP-OR; instead an Independent Trial Steering Committee (ITSC) is in place to monitor the safety and progress of the trial. The ITSC will meet approximately twice per year. The ITSC, with an independent chair, will provide overall supervision of the trial, in particular trial progress, adherence to protocol, patient safety and consideration of new information.

### Safety, discontinuation of treatment and premature termination of the trial

Safety and tolerability will include serious adverse events (SAEs) and adverse events (AEs), evaluated for grade, duration, type, onset, and relationship to study investigational medicinal product (IMP) according to the National Cancer Institute Common Terminology Criteria for Adverse Events V4.0 (NCI-CTCAE). AEs related to ofatumumab will be assessed against the Investigators Brochure (IB), and AEs related to the other drugs (non-IMPs) will be assessed against the relevant Summary of Product Characteristics (SPC). The investigator or site staff are responsible for detecting, documenting and reporting events that meet the definition of an AE or SAE to the CHOP-OR Trial Office.

Patients must receive no further treatment on trial if at either the post cycle four or six assessment, their disease response is less than a PR. Patients may be withdrawn from the trial at any time if it is the wish of the patient for any reason, if the investigator judges it necessary due to medical reasons, or if the patient becomes pregnant. Patients may withdraw consent at any time; in this event, any samples and data that have already been provided for the research trial will be retained and used in the analysis. No further samples will be taken and any surplus material will be destroyed.

The Sponsor, CHOP-OR Trial Office (OCTO) and the CI reserve the right to temporarily suspend or terminate the study at any time for reasons including (but not limited to) safety issues, ethical issues, or severe non-compliance.

### Confidentiality, dissemination of results and publication policy

Information collected during the trial is considered strictly confidential and will be securely stored with restricted access in accordance with the Data Protection Act. To protect the participant’s identity, only initials and date of birth along with an assigned trial number will be used as patient identifiers. It is clearly stated in lay terms in the patient information sheet and as a separate point within the consent form that authorised staff from the local Trust R&D office, the research team based at each study site, OCTO personnel during on site monitoring visits, and auditors from regulatory bodies may have access to their patient records.

The Sponsor recognises that participating investigators have a responsibility under the Research Governance Framework for Health and Social Care to ensure that results of scientific interest arising from the Study are appropriately published and disseminated. The Chief Investigator will ensure public disclosure of the clinical trial research results in the form of a peer reviewed scientific journal publication and for other academic research purposes as appropriate. Participating site investigators will be provided with the full summary of results and encouraged to share the summary of results with study participants, as appropriate.

### Interim analysis

To assess feasibility of recruitment, the recruitment rate is being monitored closely. If less than 10 patients had been recruited into the study within 12 months of opening the first site to recruitment the study was to be stopped because it was deemed not feasible to recruit this patient population. This milestone was passed. Next, response of the first 25 evaluable patients was assessed at the post cycle six assessment or at the time of withdrawal for those patients not completing cycle six of treatment. As seven or more patients were found to have achieved CR or PR, a further 10 patients are planned to be recruited as stated in the protocol. If any of these further 10 patients withdraw from the study and are not evaluable then they will ideally be replaced, if within the recruitment period. Statistical analyses will be undertaken using STATA/SAS.

## Discussion

The CHOP-OR trial evaluates the safety, feasibility and clinical activity of ofatumumab induction combined with CHOP and subsequent maintenance therapy in RS. The potent anti-CD20 activity of ofatumumab, together with its tolerability and efficacy in TP53 disruption provides a clear rationale for the study. The CHOP-OR study will also aim to provide further genotype-phenotype matched data to add to this growing field of interest in RS-DLBCL. Encouragingly, the interim analysis showed that seven or more patients achieved a CR or PR after induction CHOP-O and therefore the trial is continuing to full recruitment.

### Trial registration

ClinicalTrials.gov Identifier NCT01171378.

### Ethical approval

Obtained from the National Research Ethics Service (NRES) Committee South Central – Oxford A. REC reference number: 10/H0604/85. UK CLL BioBank has ethical approval to collect samples from all NCRN associated clinical studies.
